# Distribution of Important Probiotic Genes and Identification of the Biogenic Amines Produced by *Lactobacillus acidophilus* PNW3

**DOI:** 10.3390/foods9121840

**Published:** 2020-12-10

**Authors:** Kazeem Adekunle Alayande, Olayinka Ayobami Aiyegoro, Collins Njie Ateba

**Affiliations:** 1 Antibiotic Resistance and Phage Biocontrol Research Group, Department of Microbiology, North-West University, Mmabatho 2745, South Africa; collins.ateba@nwu.ac.za; 2 Food Security and Safety Niche Area, Faculty of Natural and Agricultural Sciences, North-West University, Mmabatho 2745, South Africa; 3 Gastrointestinal Microbiology and Biotechnology Division, Agricultural Research Council, Animal Production Institute, Irene 0062, South Africa; aiyegoroo@arc.agric.za; 4 Unit for Environmental Sciences and Management, North West University, Potchefstroom 2520, South Africa

**Keywords:** bioactive peptide, ornithine decarboxylase, phylogeny, insertion sequence, CRISPR-Cas

## Abstract

The genome of *Lactobacillus acidophilus* PNW3 was assessed for probiotic and safety potentials. The genome was completely sequenced, assembled using SPAdes, and thereafter annotated with NCBI prokaryotic genome annotation pipeline (PGAP) and rapid annotation using subsystem technology (RAST). Further downstream assessment was determined using appropriate bioinformatics tools. The production of biogenic amines was confirmed through HPLC analysis and the evolutionary trend of the strain was determined through the Codon Tree pipeline. The strain was predicted as a non-human pathogen. A plethora of encoding genes for lactic acids and bioactive peptides production, adhesion molecules, resistance to the harsh gut environmental conditions, and improvement of the host metabolism, which are putative for important probiotic functionalities, were located at different loci within the genome. A bacteriocin predicted to be helveticin J was identified as one of the secondary metabolites. The maximum zone of inhibition exhibited by the crude bacteriocin against STEC *E. coli* O177 was 21.7 ± 0.58 mm and 24.3 ± 1.15 mm after partial purification (250 µg/mL). Three coding sequences were identified for insertion sequences and one for the CRISPR-Cas fragment. The protein-encoding sequence for Ornithine decarboxylase was found within the genome. *L. acidophilus* PNW3 presents important features categorizing it as a viable and safe probiotic candidate, though further safety investigations are necessary. The application of probiotics in livestock-keeping would ensure improved public health and food security.

## 1. Introduction

Lactic acid bacteria (LAB) are widely used as probiotics across host species [1]. *Lactobacillus acidophilus* is a lactic acid bacterium commonly used in dairy industries as a starter culture in the production of high-quality health functional foods, such as yoghurt, cheese and beverages [2]. Complete genome sequences have contributed a great deal in the elucidation of the probiotic potentials of lactic acid bacteria [3]. Several strains of *L*. *acidophilus* have been extensively characterized and their probiotic features have been judiciously documented [4]. Studies have indicated that the bioactive secondary metabolites produced by many probiotic agents affect bacterial community interactions and potentially attenuate the disease symptoms caused by pathogens [5,6].

Probiotic yoghurts containing *L. acidophilus* improve total cholesterol and low-density lipoprotein cholesterol (LDL-C) concentrations in both male and female patients suffering from type 2 diabetes, thus suggesting a great potential use in ameliorating risk factors associated with cardiovascular diseases [7]. Surface layer proteins of the probiotic *L. acidophilus* NCFM were revealed to have a potential to protect the intestinal epithelial barrier function against TNF-α-elicited inflammation through the regulation of tight junction protein expression, the prevention of loose intestinal permeability, the blockage of cell apoptosis and the obstruction of the nuclear factor kappa light chain enhancer of the activated B cells (NF-κB) signaling pathway [8]. The presence of this promising bioactive peptide supports the use of *L*. *acidophilus* NCFM in the development of functional foods, and as a potential prophylactic agent for inflammatory bowel diseases. The surface layer protein extracted from *L. acidophilus* NCFM also induces the formation of ROS, which, in turn, results in induced auto-phagic death in HCT116 colon cancer cells through the suppression of the mammalian target of rapamycin (mTOR) activity and activation of the c-Jun N-terminal kinase signaling pathway [9]. In a study conducted by Guo et al. [10], using a rat model, it was also concluded that the administration of *L. acidophilus* CICC6074 reduced levels of inflammatory cytokines TNF-α and IL-6, intestinal apoptosis, and symptoms of Neonatal necrotizing enterocolitis.

The gut microbiota plays a crucial role in the wellbeing of the host and protection against pathogens. The mutualism between host species and their gut microbial ecosystem begins at birth, and stabilizes in humans around 3 years of age [11]. *L. acidophilus* has also been reported to be efficacious in the readjustment of impaired microbiota and the inflammation induced in Th1- (C57BL/6) and Th2 (BALB/c)-biased mice after being challenged with *Salmonella* Typhimurium at infective dose levels [12]. The aim of this study was to assess the entire genome of *L. acidophilus* PNW3 in order to determine the genome-based probiotics features which may be present in the strain.

## 2. Materials and Methods

Every procedure involved in this study complied with the relevant legislation regarding the protection of animal welfare and was approved by the Agricultural Research Council, API Ethics Committee with registration number APIEC13/008.

### 2.1. Genomic DNA Extraction and 16S rRNA Identification of the Isolate

The lactic acid bacteria strain was isolated from the gastrointestinal tracts of compassionately sacrificed weaned piglet of the indigenous South African Windsnyer pig breed (APIEC13/008), and were cultured in de Man–Rogosa–Sharpe broth (Oxoid, Hampshire, UK), under strict anaerobic conditions. A pure culture of the isolate in de Man–Rogosa–Sharpe broth was washed in phosphate buffer saline (pH 7) and the bacterial genomic DNA was extracted from the washed cells with a DNA extraction kit (Zymo Research, Irvine, CA, USA). The purity and concentration of the extracted genomic DNA were determined using a nanodrop spectrophotometer (NanoDrop 2000, ThermoFisher, Germiston, South Africa). The identification of the isolate was confirmed through PCR amplification of the 16S rRNA region [13], and the resultant PCR amplicon was sequenced. The partial sequenced data (1552 bp) were, thereafter, submitted to the GenBank data base, and an accession number (MK123485) was received (https://www.ncbi.nlm.nih.gov/nuccore/MK123485.1/).

### 2.2. Sequencing of the Entire Genome of the Isolate

Genomic DNA was prepared with an Illumina Nextera DNA flex library prep kit and the reads library was sequenced on an Illumina MiSeq instrument at the ARC, Biotechnology Platform, South Africa, to generate 3.05 GB of data. The library contains a total of 4,944,578 reads with read lengths of 2 × 300 bp paired-end. Conditions for the run and the basic statistical features of the entire genome assembly have been mentioned in our previous study [14].

### 2.3. Determination of Important Probiotic Genes

The genome of the isolate was assembled using SPAdes v. 3.12.0 [15] and was functionally annotated with NCBI prokaryotic genome annotation pipeline (PGAP) v. 4.7 [16,17] and rapid annotations using subsystems technology (RAST) with SEED viewer v. 2.0 [18]. The presence and location of the probiotic important genes was manually searched through the genome functional annotations.

### 2.4. Determination and Extraction of Bioactive Secondary Metabolite

The rapid in silico analysis to determine the presence of secondary metabolite biosynthesis gene clusters was performed using antiSMASH v. 5.0.0beta1 [19] and supported with a manual search through functional annotations.

The identified bioactive peptide (bacteriocin) was extracted after the bacterial isolate was sub-cultured into de Man–Rogosa–Sharpe (MRS) broth under complete anaerobic conditions and incubated at 37 °C for 24 h under the influence of Microbiology Anaerocult A (Merk, Darmstadt, Germany). Thereafter, the culture was centrifuged at 10,000 rpm for over 10 min and the pH of the supernatant was adjusted to 6.5 before being filtered through a 0.22 μm syringe filter (Millex syringe filters, Sigma Aldrich, Darmstadt, Germany). One liter of the filtered supernatant (crude bacteriocin) was partially purified via precipitation with 80% saturated ammonium sulphate and left overnight at 4 °C with regular stirring. The precipitates were collected and re-suspended in 0.1 M phosphate buffer solution at pH 7 and subsequently extracted in a mixture of chloroform and methanol (2:1, *v*/*v*) [20,21].

### 2.5. Susceptibility Testing of the Bioactive Peptide

The agar well diffusion method was used to determine the antimicrobial potential of the bioactive peptide produced by the probiotic bacterial isolates against pathogenic Shiga toxigenic *E. coli* (STEC) *Escherichia coli* O177 [22]. The *E. coli* O177 strains were collected from the culture collection of the Molecular Microbiology Research Laboratory, North-West University Mafikeng Campus, South Africa. A 24 h old nutrient broth culture of each isolate was standardized (0.5 McFarland) and 100 μL of the standard inoculums was inoculated into molten (50 °C) sterile Muller Hinton agar, gently mixed and then plated. The seeded agar medium was allowed to set before the wells were bored into it, using a cork borer (6 mm). The wells were carefully filled with the bacteriocin extract and incubated at 37 °C for 24 h. Thereafter, the clear zones of inhibition were measured. The experiment was carried out in three replicates.

### 2.6. Genomic Assessment of the Isolate for Safety

The entire genome of the *Lactobacillus acidophilus* PNW3 was assessed to evaluate how safe the isolate were for a probiotic candidate. PathogenFinder v. 1.1 [23] was used to determine the pathogenicity of *L. acidophilus* PNW3 towards human hosts. ResFinder v. 3.1 [24] and the Comprehensive Antibiotic Resistance Database (CARD) v. RGI 5.1.0, CARD 3.0.7 [25] were employed for the presence of antimicrobial resistance genes. The possibility of virulence determinant within the genome was assessed using VirulenceFinder v. 2.0 [26] in combination with manual searches, through the annotation, for protein-encoding sequences related to virulence in the isolate. The virulence determinants considered during the search include sex pheromones, gelatinase, cytolysin, hyaluronidase, aggregation substance, enterococcal surface protein, endocarditis antigen, adhesine of collagen and integration factors.

The rapid identification and annotation of prophage sequences within the genome of *L*. *acidophilus* PNW3 was determined using the Phage Search Tool (PHAST) [27] and the Phage Search Tool Enhanced Release (PHASTER) [28]. The genome was searched for insertion sequences (IS) using the ISfinder search tool, Insertion Sequence Semi-Automatic Genome Annotation (ISsaga V. 2.0) [29] and the Optimised Annotation System for Insertion Sequences (OASIS) [30]. Protein-encoding genes for transposase were manually searched through the functional annotation data. Coding sequences for clustered regularly interspaced short palindromic repeats (CRISPR) and the associated Cas-genes were identified using CRISPRCasFinder v. 1.1.2-I2BC [31].

### 2.7. Determination of Possible Biosynthesis of Toxic Biochemical

Protein-encoding sequences putative for the production of biogenic amines, such as ornithine decarboxylase, histidine decarboxylase, agmatine dehydrolase, L-lysine decarboxylase, tyrosine decarboxylase and the agmatine deiminase pathway, were manually investigated through the functional annotation. Other toxins that were considered in the search included haemolysin, cytotoxin K, fengygin, surfactins and lychenisin.

### 2.8. Biochemical Extraction and Determination of Biogenic Amines

The isolate was sub-cultured in de Man–Rogosa–Sharpe broth supplemented with amino acid supplements, L-histidine mono-hydrochloride (2.5 g/L), L-tyrosine disodium salt (2.5 g/L), L-ornithine mono-hydrochloride (2.5 g/L), L-lysine mono-hydrochloride (2.5 g/L) and agmatine sulfate salt (1 g/L) (Sigma-Aldrich, Darmstadt, Germany). The culture was incubated, under strict anaerobic conditions at 37 °C for 72 h without agitation [32,33]. Histamine, putrescine, agmatine, cadaverin and tyramine were extracted and determined following the procedure as previously reported by Singracha et al. [34]. Exactly 50 mL of the bacterial culture was centrifuged at 10,000 rpm over a 10 min period at 10 °C. Then, 5 mL of the supernatant thereof was extracted in 25 mL of 0.4 M perchloric acid before being transferred into a screw-capped bottle. Thereafter, 1 mL of crude extract was added with 10 μL of the internal standard (1, 7-diaminoheptane), 200 μL of 2M NaOH, 300 μL of saturated NaHCO_3_ and 1000 μL of dansyl chloride (10 mg/mL in acetone), and vortexed. The thoroughly mixed suspension was incubated for 30 min at 70 °C. The excess dansyl chloride was then precipitated with 30% NH_4_OH (100 μL). The resulting supernatant was adjusted to 50 mL with acetonitrile and filtered through a 0.45 μm Polytetrafluoroethylene membrane filter, and kept at −28 °C before the HPLC analysis.

Determination of biogenic amines from the supernatant was carried out using advanced optical detection with exceptional chromatographic and spectral sensitivity, and high-performance liquid chromatography (PerkinElmer Altus, A-10 PDA Detector) with a C18 RP-HPLC column (Analytical C18 Column 100 × 4.6 mm, 5 μm particle size). The combination of acetonitrile and deionized water was used as the gradient elution system, while 0.5 mL/min was set as the flow rate. The elution gradient of 65% acetonitrile was set at 0 min, and was increased to 70% at 5 min and 100% at 20 min, and then 65% at 25 min. Histamine, tyramine, cadaverine, putrescine and L-agmatine (Sigma-Aldrich, Darmstadt, Germany) were used as standards.

### 2.9. Shared Protein-Based Phylogeny between L. acidophilus PNW3 and Other Strains Isolated from Different Locations and Sources

The phylogeny was developed using the Codon Tree pipeline host on the PATRIC v3.6.6 [35]. The pipeline uses amino acids and nucleotide sequences from a defined number (500) of PATRIC’s global protein families (PGFams) [36]. These were randomly picked to build an alignment and then generate a tree based on the differences among the selected coding sequences. The protein-encoding sequences were aligned using MUSCLE [37], while the nucleotide coding gene sequences were aligned using the codon align function of BioPython [38]. A concatenated alignment of all proteins and nucleotides was written into a phylip-formatted file, and then we generated a partitions file for RaxML [39]. The support values were generated using 100 rounds of the “Rapid” bootstrapping option [40] of RaxML. The resulting newick file was downloaded and viewed in the FigTree [41].

## 3. Results

**Figure 1:**
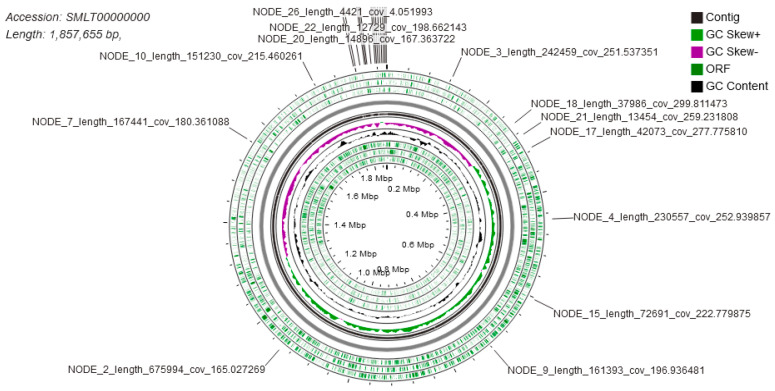
Circular genome mapping with position of each contig within the *L. acidophilus* PNW3 genome. The mapping was generated using CGView [42].

The sequenced set of reads and complete genome assembly of the *Lactobacillus acidophilus* PNW3 has been deposited in SRA and the DDBJ/ENA/GenBank under the accession numbers SRX5395058 and SMLT00000000, respectively. The entire genome, assembled in 25 contigs, contains 1776 coding sequences. Figure 1 shows a circular representation of the genome with the relative position of each of the contigs.

### 3.1. Important Probiotic Genes

The probiotic features determined within the genome include coding sequences putatively involved in the following: production of lactic acids; production of bioactive peptides; production of adhesion molecules; production of extracellular enzymes; development of stress resistance mechanism; and stimulation of active metabolism in the host (Table 1). Three protein-encoding sequences were determined for L-lactate dehydrogenase (EC 1.1.1.27), which functions in the fermentation of lactate and mixed acids. Six different coding sequences putatively involved in the biosynthesis of bacteriocin were identified within the genome of the *L. acidophilus* PNW3. One coding sequence was determined encoding for FIG006988 (Lipase/Acylhydrolase with GDSL-like motif (390 bp)) and three were found encoding for Esterase/Lipase, which are 795, 822 and 897 base-pairs long, respectively. Twelve protein-encoding sequences were also found for different forms of extracellular protease, while none were identified for both amylase and cellulase.

### 3.2. Efficacy of the Bioactive Secondary Metabolites

**Figure 2:**
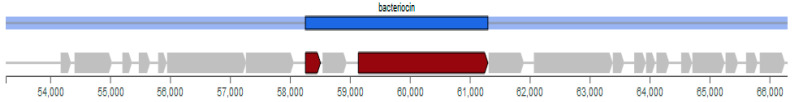
Gene cluster showing the position of biosynthetic bacteriocin. The core biosynthetic (bacteriocin) genes (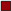), other genes (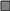), and the locations of the gene clusters were mapped by antiSMASH v5.0.0beta1.

Both the manual search through the functional annotation and the rapid in silico analysis for secondary metabolite biosynthesis gene clusters revealed the presence CDS, predicted for bacteriocin (Figure 2). The bacteriocin extracted from the *Lactobacillus acidophilus* PNW3 was partially purified and tested against two different strains of Shiga toxin-producing *E. coli* O177. Both strains were susceptible to the crude and partially purified bacteriocin with different ranges of zone of inhibition (Table 2).

### 3.3. Safety Assessment of the Strain

Both the ResFinder and CARD database for the genes encoding for acquired drug resistance revealed the presence of Tetracycline-resistant ribosomal protection protein (*tetW*) and Lincosamide nucleotidyltransferase (*lnuC*), which confer resistance to Tetracycline and Lincosamide, respectively. Manual searches through the functional annotation only indicate the presence of multidrug-resistant proteins with assisting heterodimetric efflux ABC transporters (LmrC subunit of LmrCD). The strain was predictably identified as a non-human pathogen by the PathogenFinder tool hosted by the Centre for Genomic Epidemiology. The calculated Matched Pathogenic Families was 0, while the Matched Not Pathogenic Families was 597, and the probability of being a human pathogen was calculated to be 0.2. There was no hit for virulence determinants using VirulenceFinder.

**Figure 3:**
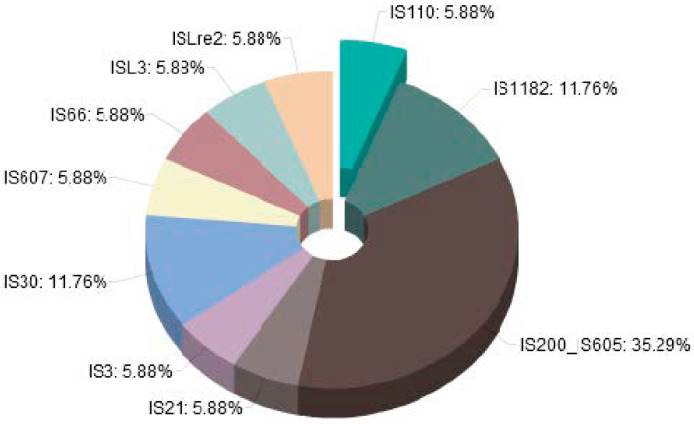
Genome mapping of the *L. acidophilus* PNW3 showing the distribution of the roughly predicted IS family within the genome using the ISsaga v2.0.

A handful of Mobile Genetic Elements were equally identified within the genome of the *Lactobacillus acidophilus* PNW3. A single prophage region was identified within the entire genome, and the coding sequence indicates incomplete prophage type. Five coding sequences were also predicted for insertion sequences (IS), with three different identified IS distributed into three different families using the Optimised Annotation System for Insertion Sequences search tool. The associated IS families were S200–IS605, IS30 and IS66, while on the other hand, 10 putative complete open reading frames (ORF) putative for IS were identified using the ISsaga search tool and distributed into 10 different families (Figure 3).

A search for the CRISPR-Cas sequence within the *L. acidophilus* PNW3 genome revealed only one CDS putative for the CRISPR sequence with the associated cas gene. The fragment occurred on Contig Identity NODE_10_length_151230_cov_215_460261_1 and occupied the region between 64,572 and 66,430 bp. The identified CRISPR sequence contained 30 spacer genes and a 28 bp repeat consensus (GGATCACCTCCACATACGTGGAGAAAAT).

### 3.4. Detection of Biogenic Amines Produced by the Strain

**Figure 4:**
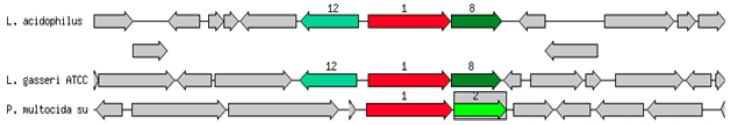
Annotation diagram mapping out the location of Ornithine decarboxylase (EC 4.1.1.17) (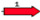) found within the *L acidophilus* PNW3 genome.

Only one protein-encoding sequence related to the production of biogenic amines was found within the entire genome of the *L. acidophilus* PNW3. The CDS is putative for Ornithine decarboxylase (EC 4.1.1.17) and located on Contig Identity NODE_2_length_675994_cov_165.027269. The length of the fragment measures 2094 bp and stretches between 368,158 and 370,251 bp along the forward DNA strand (Figure 4). No hit was found for the enteroxins and lipopeptides within the genome. Analysis of the HPLC carried out on the extracted biogenic amines produced by the isolate also revealed putrescine as the only biogenic amine produced. The putrescine produced by the *L. acidophilus* PNW3 was eluted within a 2.337 min retention time, while the putrescine used as the standard reference eluted at 2.436 min under the same flow rate of 0.5 mL/min.

### 3.5. Evolutionary Similarity between the L. acidophilus PNW3 and Other Strains

**Figure 5:**
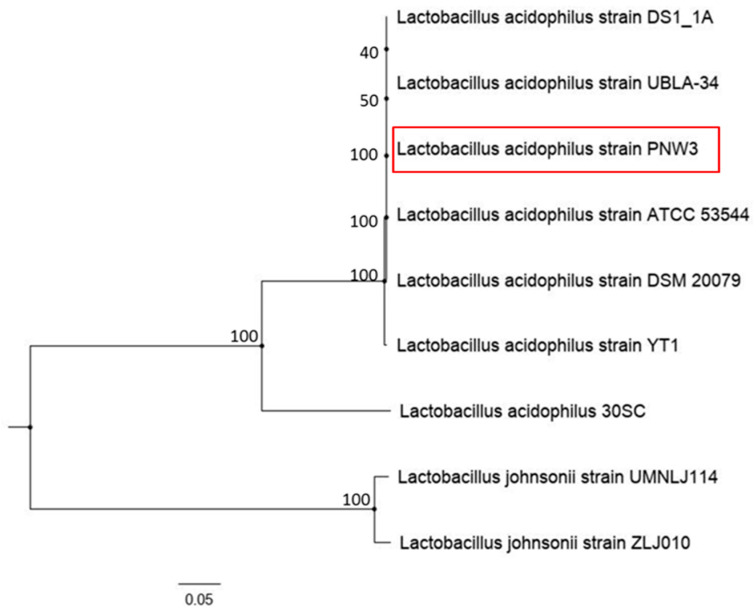
Shared protein-based phylogeny between *Lactobacillus acidophilus* PNW3 and other strains.

A total of seven genomes of different strains of *L. acidophilus*, including PNW3 and two genomes of *L. johnsonii*, were used to generate a phylogenetic tree. *L. acidophilus* PNW3 (South Africa) and 30SC (South Korea) were both isolated from *Sus scrofa*, while the ATCC 53,544 (USA), DSM 20,079 (France) and DS1_1A (USA) were isolated from *Homo sapiens*. The strain YT1 (South Korea) was from *Rattus norvegicus* and UBLA-34 (India) was from fermented food. The *Lactobacillus johnsonii* strains UMNLJ114 (USA) and ZLJ010 (China) were isolated from *Meleagris gallopavo* and *Sus scrofa*, respectively (Figure 5). The tree was built on 500 single copy genes that included 200,641 amino acids and 601,842 nucleotides. All the *L. acidophilus* appeared on the same clade, and likewise the two strains of the *L. johnsonii* had a 100% support value for each clade. Among the *L. acidophilus*, only the 30SC does not cluster with the others. The *L. acidophilus* PNW3 appears to have evolved around the same time as the other five strains, regardless of the year of isolation, location and the source of the isolates. This is supported by a 100% confidence level, except for the UBLA-34 and DS1_1A, which are 50% and 40%, respectively.

## 4. Discussion

This study is an overhaul of the entire genome of *Lactobacillus acidophilus* PNW3 for important probiotic features and the safety of the isolate. Functional annotation through the NCBI prokaryotic genome annotation pipeline and rapid annotations using subsystems technology with SEED viewer revealed several inherent protein-encoding sequences predictably in support of the health benefit of a typical probiotic candidate.

Three protein-encoding genes were determined within the genome, putative for L-lactate dehydrogenase (EC 1.1.1.27), an important enzyme required in the fermentation of lactate and mixed acids. In the in vitro and in vivo studies conducted by Yang et al. [43], lactic acid was confirmed as an inducer of the rapid de-phosphorylation of the epidermal growth factor receptor. It also exhibited inhibitory effects on the production of IL-8 induced by IL-1β, and colony formation by HT29 cells. Furthermore, lactic acid commendably increased the rate of survival when administered through the oral route to ApcMin mice (Apc/multiple intestinal neoplasia) with advanced stage malignant tumor. In another independent study, L-lactate dehydrogenase was found to possess the ability to stimulate the bioenergetics of the mitochondria in the heart, muscle and liver, just as effectively as pyruvate. The enzyme L-lactate dehydrogenase was also confirmed to stimulate the production of reactive oxygen species in the mitochondrion at the same rate as pyruvate induces the production of hydrogen peroxide [44].

Moreover, among the probiotic important genes found within the genome of *L*. *acidophilus* PNW3 are genes putative for bioactive peptides. The coding sequences for the bacteriocin, Autoinducer-2 production protein, type 1 capsular polysaccharide biosynthesis protein and mannosyltransferase involved in polysaccharide biosynthesis were found within the genome. Bioactive peptides, synthesized as secondary metabolites by the lactic acid bacteria, provide a source of alternative bioactive compounds of natural origin to synthetic antimicrobials. Several studies have revealed the potency of the active compounds secreted by LAB against clinically important pathogens and malignant cells [45,46,47].

The effectiveness of a probiotic is largely enhanced by its ability to adhere and competitively colonize gut epithelia cells. Sortase A, which is LPXTG-specific, identified with heme, hemin uptake and utilization systems in Gram-positive Sortase, and is among the important adhesion players in the *L*. *acidophilus* PNW3. Sortase A is an enzyme used by Gram-positive bacteria to coat the cell surface with functional proteins and a pili assemblage that enables interactions between the cells and their environment. The enzyme is highly important in the cell physiology and defense; it is made up of protein domains connected with the adhesion of cells to host cells and extracellular matrix proteins [48,49]. Other important adhesive encoding genes identified include the following: cell surface and S-layer proteins; fibronectin/fibrinogen-binding protein; and members of the EPS cluster such as EpsC, EpsD, EpsJ and ATP synthase epsilon chain.

The gut microbiota vested with diverse enzymatic potentials plays a crucial role in the improvement of the host metabolism, the repression of pathogens, the contribution of additional nutrients and the improvement of nutrient digestibility and absorption [50,51]. Extracellular enzymes, such as lipase, protease and amylase, among others, produced by most lactic acid bacteria, play an important role in the guts of animals and human beings, including infants [52]. Four coding sequences putative for different kinds of lipases and twelve coding sequences putative for different proteases were among the probiotic supportive genes located in the genome of *L*. *acidophilus* PNW3. This, therefore, supports the envisaging potential of the isolate with regard to improving nutrient digestibility in the host as a probiotic candidate. This may directly translate into growth performance improvement in the target farm animal.

In addition, the functional annotation of the entire genome assembly also revealed a plethora of coding genes putative for stress tolerance within the gut environment in relation to bile salts and gastric pH. These include ATP synthase epsilon chain, PTS system, cellobiose-specific IIC component, ATP-dependent Clp protease ATP-binding subunit, and L-lactate dehydrogenase, all of which are responsible for the maintenance of acid stress resistance [3]. Autoinducer-2 production protein *LuxS*, which is among the stress-resistant CDS identified in the genome, has been described as part of the proteins involved in both acid and bile salt stress tolerance [53]. Another CDS identified along the cell envelope subcategory is the cell envelope-associated transcriptional attenuator LytR-CpsA-Psr, subfamily F2. The hydrophobic nature of the cell envelope of microorganism assists with the adhesion of the organisms to the host epithelia cell, therefore conferring a competitive edge for colonization of the gastrointestinal tract [54].

Quick searches through different databases and a manual search through functional annotation revealed that *L. acidophilus* PNW3 harbors resistant genes against Lincosamide and Tetracycline. Functional annotation also revealed the presence of the heterodimeric efflux ABC transporter, and multidrug resistance. Although several heterodimeric ABC transporters have been reportedly involved in multidrug efflux in Gram-positive organisms, it is worthy to note that most of the annotated multidrug efflux transporters are not capable of transporting drugs, but rather get involved in other physiological functions within the cell [55]. Moreover, the heterodimeric efflux ABC transporter is an intrinsic form of resistance, which has little or no chance of being transferred to other bacteria [56]. The only prophage region found within the *L. acidophilus* PNW3 genome was designated as an incomplete prophage type, an indication of its non-functionality. None of the identified transposase and other insertion sequences flanked the resistance genes, thus further limiting the transferability of these antimicrobial resistance genes.

Furthermore, the genome of the isolate harbors a fragment of clustered regularly interspaced short palindromic repeats (CRISPR) with associated Cas-gene and spacer. The presence of a CRISPR region within the microbial genome is expected to limit the spread of antimicrobial resistant genes through the obstruction of multiple pathways of lateral gene transfer [57]. The CRISPR-Cas fragment equips the host strain with a sequence-specific defense-line against the intrusion of extra-chromosomal DNA molecules such as insertion sequences (IS), plasmids, transposons and phages [58,59]. The presence of this therefore indicates the stability of the *L. acidophilus* PNW3 genome and the reduced possibility of acquiring antimicrobial-resistant genes.

The CDS putative for ornithine decarboxylase (EC 4.1.1.17) was the only protein with the potential for producing a biogenic amine compound in the *L. acidophilus* PNW3. The decarboxylation of amino acid ornithine by the enzyme ornithine decarboxylase leads to the formation of putrescine, which is a biochemical that has been confirmed to down-regulate the activities of macrophages in response to injury due to microbial infection [60]. The production of putrescine by the *L. acidophilus* PNW3 was practically confirmed through HPLC analysis. Ornithine decarboxylase also limits the rate of polyamine biosynthesis, thus increasing the potential for oncogenesis [61]. The increased activity of the enzyme has been reportedly observed in the rapid proliferation of normal and neoplastic cells. Although the activity of Ornithine decarboxylase is under strict regulation at transcriptional and post-translational stages, due to its impact on the cellular processes [62], Ornithine degradation is regulated by a protein known as antizyme in response to the density of polyamine, and is also one of the targets of the Myc/Max transcription factor [63].

## 5. Conclusions

The genome of *L. acidophilus* PNW3 is vested with enough protein-encoding genes in support of its probiotic efficacy, and the organism appears safe at the genome level. Neither of the two identified drug resistance genes were flanked by the insertion sequences, which thus reduces any potential risk of the resistant gene transferring into the environment; this could also be prevented by the presence of the CRISPR-Cas fragment. In general, *L. acidophilus* PNW3 is confirmed as a non-human pathogen, though the strain is capable of producing putrescine, which is a biogenic amine. Further in vivo assessment is required on the safety assurance of the strain as a viable probiotic agent. The application of probiotics in livestock keeping would ensure improved public health and food security.

*NB*. The data discussed in this article are part of the thesis submitted to the repository of North-West University, South Africa (repository.nwu.ac.za).

## Figures and Tables

**Table 1:** Protein-encoding sequences putative for important probiotic functions.

Important Probiotic Features	Associated Functional Proteins/Peptides	Gene Size (bp)	CDS Contigs Identity
Production of lactic acids	L-lactate dehydrogenase (EC 1.1.1.27)	915	NODE_10_length_151230_cov_215.460261
		927	NODE_2_length_675994_cov_165.027269
		972	NODE_4_length_230557_cov_252.939857
Production of bioactive peptides	Bacteriocin helveticin J	978	NODE_10_length_151230_cov_215.460261
	Bacteriocin pre-peptide/inducing factor for bacteriocin synthesis	162	NODE_3_length_242459_cov_251.537351
		192	NODE_3_length_242459_cov_251.537351
	Three-component quorum-sensing regulatory system, inducing peptide for bacteriocin biosynthesis	144	NODE_3_length_242459_cov_251.537351
	Bacteriocin ABC-transporter, ATP-binding and permease component	2163	NODE_3_length_242459_cov_251.537351
		591	NODE_3_length_242459_cov_251.537351
	S-ribosylhomocysteine lyase (EC 4.4.1.21) @ Autoinducer-2 production protein LuxS	474	NODE_2_length_675994_cov_165.027269
		156	NODE_2_length_675994_cov_165.027269
	Type 1 capsular polysaccharide biosynthesis protein (EC:2.4.1.-)	702	NODE_2_length_675994_cov_165.027269
	Mannosyltransferase involved in polysaccharide biosynthesis	699	NODE_9_length_161393_cov_196.936481
Production of adhesion molecules	Sortase A, LPXTG specific	690	NODE_2_length_675994_cov_165.027269
	Cell surface protein, ErfK family	609	NODE_2_length_675994_cov_165.027269
		690	NODE_10_length_151230_cov_215.460261
	Cell surface protein precursor	1932	NODE_10_length_151230_cov_215.460261
	Cell surface protein	1020	NODE_7_length_167441_cov_180.361088
	Fibronectin/fibrinogen-binding protein	1692	NODE_2_length_675994_cov_165.027266
	S-layer protein precursor	1500	NODE_9_length_161393_cov_196.936481
	ATP synthase epsilon chain (EC 3.6.3.14)	441	NODE_2_length_675994_cov_165.027269
	Tyrosine-protein kinase transmembrane modulator EpsC	876	NODE_3_length_242459_cov_251.537351
	Tyrosine-protein kinase EpsD (EC 2.7.10.2)	783	NODE_3_length_242459_cov_251.537351
	“COG1887: Putative glycosyl/glycerophosphate transferases involved in teichoic acid biosynthesis TagF/TagB/EpsJ/RodC/Putative polyribitolphosphotransferase/CDP-ribitol: poly (ribitol phosphate) ribitol phosphotransferase/CDP-glycerol: poly (glycerophosphate) glycerophosphotransferase (EC 2.7.8.12)/CDP-glycerol: N-acetyl-beta-D-mannosaminyl-1,4-N-acetyl-D-glucosaminyldiphosphoundecaprenyl glycerophosphotransferase”	1155	NODE_9_length_161393_cov_196.936481
Production of stress resistance molecules	Cell envelope-associated transcriptional attenuator LytR-CpsA-Psr, subfamily F2	1275	NODE_3_length_242459_cov_251.537351
		1056	NODE_3_length_242459_cov_251.537351
		1104	NODE_4_length_230557_cov_252.939857
	ATP-dependent Clp protease, ATP-binding subunit ClpE	2187	NODE_2_length_675994_cov_165.027269
	ATP-dependent Clp protease, ATP-binding subunit ClpC	2478	NODE_4_length_230557_cov_252.939857
	Peptide-methionine (R)-S-oxide reductase MsrB (EC 1.8.4.12)	438	NODE_7_length_167441_cov_180.361088
	S-ribosylhomocysteine lyase (EC 4.4.1.21) @ Autoinducer-2 production protein LuxS	474	NODE_2_length_675994_cov_165.027269
		156	NODE_2_length_675994_cov_165.027269
Improving host metabolism	2-diacylglycerol and 3-glucosyltransferase (EC 2.4.1.337)	1164	NODE_20_length_14896_cov_167.363722
	Poly (glycerol-phosphate) alpha-glucosyltransferase (EC 2.4.1.52)	1092	NODE_9_length_161393_cov_196.936481
	Cellobiose phosphor transferase system celC	447	NODE_22_length_12729_cov_198.662143
	PTS system, cellobiose-specific IIC component	1326	NODE_2_length_675994_cov_165.027269
	PTS system, cellobiose-specific IIB component (EC 2.7.1.205)	336	NODE_2_length_675994_cov_165.027269
	Outer surface protein of unknown function, cellobiose operon	1062	NODE_3_length_242459_cov_251.537351
	Methionine synthase II (cobalamin-independent)	1119	NODE_2_length_675994_cov_165.027269

**Table 2:** Susceptibility test of crude and partially purified bacteriocin produced *L. acidophilus* PNW3.

Organisms	Zone of Inhibitions (mm) **	
	Crude Bacteriocin	Partially Purified Bacteriocin (250 µg/mL)
*E. coli* C1	21.7 ± 0.58	24.3 ± 1.15
*E. coli* C2	19.3 ± 0.58	24.0 ± 1.00

Key: **—Mean values of three replicates.
